# Pregnancy associated atypical hemolytic uremic syndrome presenting with preeclampsia with HELLP syndrome and following treatment with Eculizumab

**DOI:** 10.1515/crpm-2022-0016

**Published:** 2022-12-19

**Authors:** Fery Gunawan, Mandy Mangler, Cindy Sanders, Trisha Ardine Leonardo, Yosefina Cindy

**Affiliations:** Department of Obstetric and Gynecology, Hospital Werner Forßmann, Eberswalde, Germany; Department of Obstetric and Gynecology, Hospital Augusto Viktoria, Berlin, Germany

**Keywords:** atypical hemolytic uremic syndrome, Eculizumab, HELLP, microangiopathy, preeclampsia, pregnancy

## Abstract

**Objectives:**

Pregnancy associated atypical hemolytic uremic syndrome (p-aHUS) is a rare condition of thrombotic microangiopathy (TMA) which causes an increase of fetal and maternal morbidity and mortality. It presents typically with a triad of microangiopathic hemolytic anemia (MAHA), thrombocytopenia and acute progressive renal failure. Differential diagnoses of HELLP (Hemolysis, Elevated Liver enzymes, and Low Platelets) syndrome, preeclampsia, thrombotic thrombocytopenic purpura (TTP), and disseminated intravascular coagulation (DIC) syndrome must be considered. In the following case report, presented is a 32-year-old, 38 weeks pregnant Caucasian woman admitted to Eberswalde Hospital with signs of preeclampsia and HELLP Syndrome. Caesarean Section was performed due to HELLP syndrome and fetal distress. Acute renal failure occurs shortly after a successful delivery. After a diagnosis of p-aHUS is established, the patient was given Eculizumab, which yielded significant improvements.

**Case presentation:**

A 32-year-old, 38 week pregnant Caucasian woman was admitted to Eberswalde Hospital with upper right abdominal pain. After a laboratory examination, a diagnosis of HELLP syndrome was established and a Caesarean Section was performed. The follow-up examination revealed deterioration of clinical signs with the patient experiencing dyspnea, oliguria, and oedema, as well as aggravation of laboratory values, ranging from severe thrombocytopenia, hemolytic anemia, liver injury, and acute kidney injury. After excluding other possible causes of TMA, a diagnosis of p-aHUS was established and a treatment with Eculizumab was administered. Clinical and laboratory signs of hemolysis and kidney functions were found to improve gradually after two administrations of Eculizumab. The patient was discharged after 20 days of hospitalization with significantly improved condition and hematological values.

**Conclusions:**

A successful treatment of p-aHUS requires a comprehensive assessment and a prompt diagnosis, which can be confounded by multiple similar differential diagnoses. Treatment with Eculizumab was found to significantly improve the outcome of the patient, but more studies are required to decide on a standardized regiment for p-aHUS.

## Introduction

Hemolytic uremic syndrome (HUS) is a rare thrombotic microangiopathy (TMA) characterized with a triad of microangiopathic hemolytic anemia (MAHA), thrombocytopenia and significant renal dysfunction. It is usually found in children and caused by infectious agents, most commonly Shiga toxin bearing *Escherichia coli* (STEC-HUS) but also Shigella and *Streptococcus pneumoniae*. The overall incidence of HUS is estimated to be 1 to 2 cases per 100,000, but no infectious cause is found in 5–10% of the patients, which then are classified as atypical hemolytic uremic syndrome (aHUS) [1, 2]. AHUS is an extremely rare genetic disorder precipitated by unopposed complement activation, which leads to the overactivation of alternative complement pathway, causing systemic TMA and endothelial damage [1, 3, 4]. Unlike HUS, the clinical outcome of aHUS is poor, with mortality rates as high as 25% during acute episodes and up to 60% of the case progressing to end stage renal failure (ESRF) [2, 5].

Pregnancy is a well-recognized trigger for aHUS, which birthed the term pregnancy associated atypical hemolytic uremic syndrome (p-aHUS). The incidence rate was estimated to be one in every 25,000 pregnancies and 20% of all cases of aHUS in women [6, 7]. This rare, progressive, life-threatening condition can occur in all stages of pregnancy but mostly during the postpartum period [8, 9]. P-aHUS can be difficult to distinguish from thrombocyte thrombocytopenic purpura (TTP), HELLP (Hemolysis, Elevated Liver enzymes, and Low Platelets) Syndrome, preeclampsia, and other hypertensive disorders of pregnancy due to the similar TMA mechanism [9], [10], [11].

Historically, aHUS was managed with plasma infusion (PI) and/or exchange (PE). However, the efficacy has been found to be limited in various reports, with a high rate of progression to ESRF [3, 4, 7, 10, 12], [13], [14], [15]. Alternatively, Eculizumab, a human monoclonal antibody against complement protein C5, has been approved by the United States Food and Drug Administration (FDA) since 2011 and is now widely used as treatment for aHUS as well as paroxysmal nocturnal hemoglobinuria (PNH) [2, 13, 14]. The use of Eculizumab has exhibited significant improvement of outcome as compared to PE in prospective clinical trials and in several published cases for the treatment of p-aHUS [13], [14], [15], [16].

In this case study we describe a patient who was initially presented with HELLP syndrome but then revealed to have p-aHUS and successfully treated with Eculizumab.

## Case presentation

A 32-year-old 38 week-pregnant Caucasian female was admitted to the emergency department with right upper abdominal pain since 2 weeks prior, which suddenly worsened 2 h before admission. She had been diagnosed with Gestational Hypertension and treated with Methyldopa. Initial examination during admission revealed elevated blood pressure of 180/110 mmHg and proteinuria with urinary dipstick test (3+). Laboratory investigation revealed mild thrombocytopenia (125.000/uL), increased alanine-aminotransferase (ALT) level of 10.54 μkat/L (=632 U/L) and aspartate-aminotransferase (AST) level of 13.20 μkat/L (=792 U/L), increased lactate dehydrogenase (LDH) levels of 28.10 μkat/L (=1686 U/L), hypoalbuminemia (25.0 g/L), and high serum folate (>20.0 ng/mL). An ultrasound scan of the fetus revealed normal development, with weight estimation at the 5 SD growth curve. Cardiotocography (CTG) examination resulted in pathologic CTG. Diagnosis of preeclampsia with HELLP syndrome was made based on clinical and biochemical features.

Due to the deteriorating condition of the patient and fetal distress, an emergency Caesarean section was performed. A female newborn was delivered with respiratory distress, weighing 2,466 g at 5 SD, APGAR Score of 0/2/5, positive Rhesus, umbilical artery pH value of 7.06 and BE −9.1. The patient was then admitted to intensive care. Within 4 h postpartum, a hypertensive crisis, rapidly progressive severe anemia with thrombocytopenia, as well as liver and renal impairment was recorded. Hemoglobin level went down to 5.7 g/dL, platelet count dropped to 57 × 10^9^/L, haptoglobin level dropped to <7.4 mg/dL, serum creatinine went up to 259 μmol/L, urine excretion deteriorated to around 7 mL/h. With the presence of thrombocytopenia, anemia with fragmentation of red blood cell (RBC) in blood film and evidence of hemolysis, TMA was considered. A disintegrin and metalloproteinase with a thrombospondin type 1 motif, member 13 (ADAMTS13) levels were sent for examination and daily plasma exchange therapy was initiated.

The patient’s blood pressure was managed and maintained with intravenous antihypertensive agents and she was given prophylactic anticonvulsant therapy with Magnesium. Due to ongoing hemolytic anemia, she was given multiple transfusion of blood products: four (4) units of red blood cell for four (4) days, three (3) units of fibrinogen and three (3) units of human prothrombin complex. The patient also underwent plasmapheresis with 42 units of fresh frozen plasma (FFP) for three (3) days. Rhophylac (Anti-D IgG 1500 IE) was also given due to Rhesus incompatibility with her baby.

Renal sonography revealed patent renal vasculature with no blockage. Initial chest X-ray revealed basal pleural effusion which then progressed significantly over the next four days. Thoracocentesis was performed due to progressive dyspnea, yielding 500 mL of serous fluid. Transthoracic echocardiography results revealed normal functions of the left and right ventricle with no valve abnormalities.

Further diagnostics were made for differential diagnostic purposes. Complement tests revealed alternative pathway dysregulation with low plasma level of C3 at 47.1 mg/dL (90–180 mg/dL) and normal level of C4 at 12.5 mg/dL (10–40 mg/dL). Genotyping with Sanger sequencing of CFH and CFI genes showed no abnormality. Autoimmune diagnostic tests revealed negative Antinuclear Antibodies (ANA), c-ANCA, p-ANCA Serum. ADAMTS13 activity was within normal range (53%), which excluded TTP. Stool sample showed a negative test for *E. coli* and the patient had no previous history of diarrhea, excluding STEC-HUS. HELLP syndrome was doubted due to lack of significant postoperative liver function improvement.

**Figure 1:**
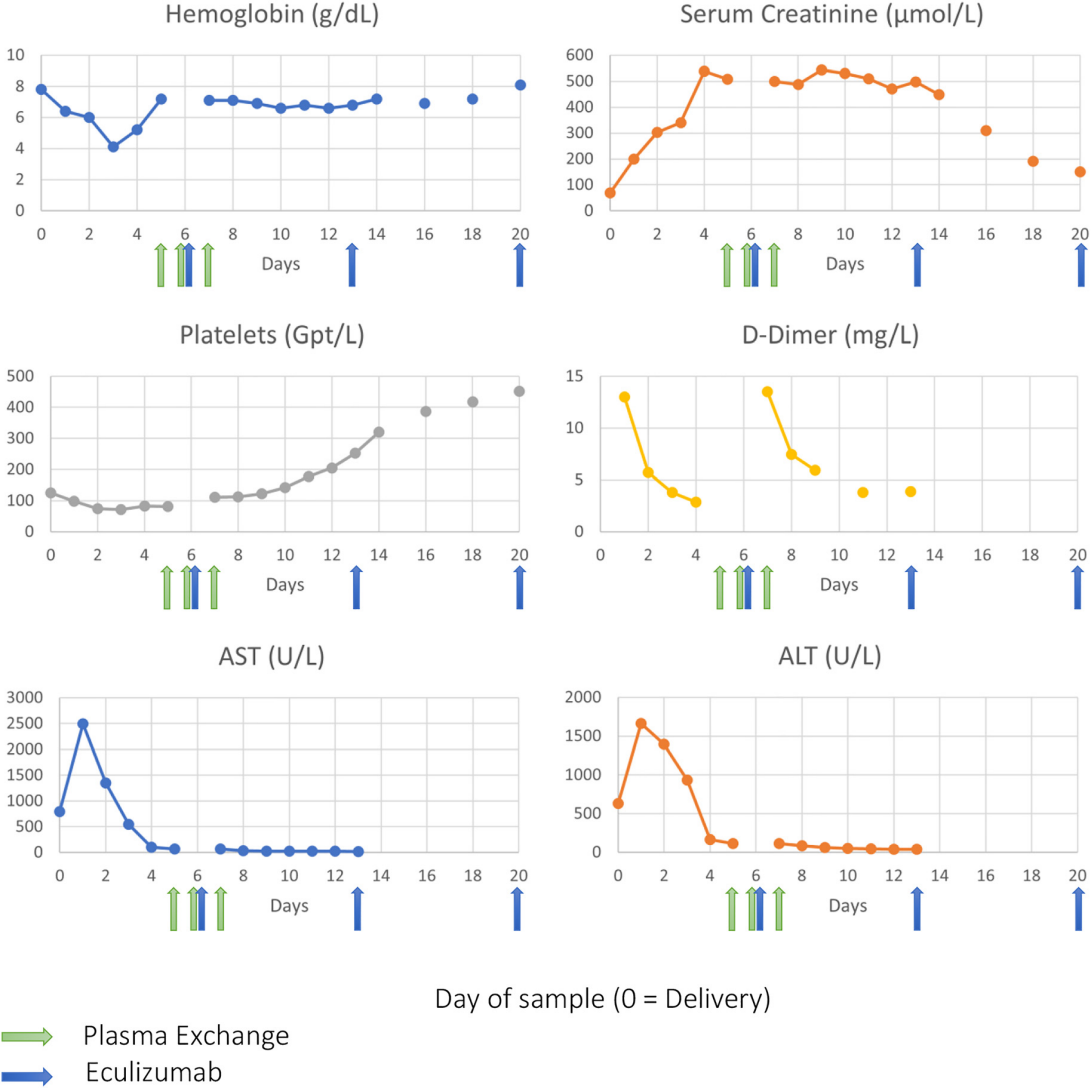
Development of selected laboratory values during the early stages of illness with administration of plasma exchange and Eculizumab.

Based on our suspicion of p-AHUS, the patient then received prophylactic antibiotic therapy and vaccinated against meningococcus before a therapy of Eculizumab 900 mg was initiated. The patient’s clinical condition improved significantly after the first dose of Eculizumab, with stabilizing blood pressure and the resolution of dyspnea and hemolysis, as well as a gradual restoration of hematologic parameters, renal, and liver function (Figure 1). After being treated for 14 days in the Intensive Care Unit (ICU), the patient‘s condition stabilized and she was transferred to the nephrology department for further management.

## Discussion

This case highlights the importance of early precise diagnosis and treatment of pregnancy-associated atypical Hemolytic Uremic Anemia (p-aHUS). It may be difficult to distinguish p-aHUS from other thrombotic microangiopathy (TMA) such as thrombotic thrombocytopenic purpura (TTP) or other pregnancy-triggered diseases related to complement activation such as HELLP syndrome [1, 13, 16].

Our patient was first diagnosed with HELLP syndrome based on initial laboratory parameters of hemolysis, elevated liver enzymes, and thrombocytopenia. After a successful emergency Caesarean section, the patient’s condition rapidly deteriorated, with progressive severe anemia, kidney function impairment, as well as no improvements in liver function. Patients with HELLP syndrome typically recover in the postpartum period and demonstrate predominantly hepatic rather than renal impairment [11]. However, due to oliguria and rapidly worsening kidney functions in the postpartum period, p-aHUS was strongly suspected [10].

The follow up laboratory values also showed signs of progressive hemolytic anemia, thrombocytopenia, and fragmentation of red blood cell in blood film. Therefore, TMA was considered, plasma exchange therapy was started and a sample for ADAMTS13 examination was sent to determine between aHUS and TTP. Clinical presentations of aHUS are often indistinguishable from TTP. However, it has been established since 1998 that TTP is caused by severe deficiency of ADAMTS13 (von Willebrand factor-cleaving metalloprotease) due to autoimmune antibodies. Thus TTP is recognized as a distinct disorder with low to no detectable levels of ADAMTS13 activity as the defining feature of TTP [1, 8]. Historically, TTP and aHUS are treated similarly with Plasma Exchange (PE), which yields excellent prognosis for TTP but not for aHUS [9]. Due to normal ADAMTS13 levels in our case, the diagnosis of TTP was excluded.

Furthermore, Shiga toxin producing *E. coli* hemolytic uremic syndrome (STEC-HUS) usually occurs up to 90% in children and can be recognized by diarrheal symptoms and discovery of *E. coli* in stool sample [1, 2, 16]. Therefore, negative test in our patient’s stool ruled out this diagnosis.

aHUS is a life threatening, rare genetic disorder. It is characterized by anemia, thrombocytopenia, and renal impairment, resulting from dysregulation of the alternative complement pathway [1, 10]. A few studies have shown genetic predisposition in aHUS. Mutation in genes that are responsible for complement regulation may cause uncontrolled activation of the complement cascade, which may lead to aHUS [8]. There are currently several identified gene mutations associated with complement regulatory proteins such as factor H, factor I, membrane cofactor protein (MCP/CD46), factor B, and factor C3 [7, 8, 10]. In this case, the patient has no detected mutations in complement regulatory gene, but presented with a known risk factor, which is pregnancy complicated with preeclampsia and HELLP syndrome. Certain risk factors known as Complement Amplifying Conditions (CACs) increase the risk of developing aHUS through activation on the alternative complement pathway which leads to microangiopathy and endothelial damage. Some of the known CACs include pregnancy complications such as preeclampsia and HELLP syndrome, autoimmune diseases, malignant hypertension, and organ transplantation [1, 16]. Pregnancy-associated hemolytic uremic syndrome occurs mostly during postpartum period, which may be stimulated by inflammatory responses, migration of fetal cells into the maternal circulation, infection, and hemorrhage [8, 16].

Eculizumab is a humanized monoclonal antibody against complement protein C5 which since 2011 has been approved by the Food and Drug Administration (FDA) and the European Medicines Agency (EMA) as the treatment for aHUS as well as paroxysmal nocturnal hemoglobinuria (PNH). Furthermore, successful use of Eculizumab has also been reported for various off-label indications such as antiphospholipid syndrome (APS), sickle cell disease (SCD) and HELLP syndrome, all of which involve excessive complement activation in the pathogenesis [2, 11, 12]. The most common side-effect of Eculizumab treatment is headache, while serious life-threatening complications such as meningococcal infections are uncommon. Patients should be vaccinated against meningococcus at least 2 weeks prior to treatment, or with appropriate prophylactic antibiotics until 2 weeks after vaccination [2, 5, 17]. Furthermore, no cases of serious neonatal infectious morbidity have been reported, and Eculizumab was not found to cross the placenta in a high enough level to affect complement activation in the fetus nor was it detected in umbilical cord nor neonatal blood samples as well as breast milk [5, 7].

Previously, women with a history of aHUS were discouraged from pregnancy due to the difficulty in management and poor prognosis. With the advance of Eculizumab therapy, there is now a better chance to maintain a pregnancy successfully [4, 5], although a relapse has also been reported [3]. A systematic review by Gupta, et al. in 2020 suggested that women diagnosed with aHUS or p-aHUS with chronic kidney disease (CKD) (serum creatinine ≥1.5 mg/dL) have a poor prognosis for future pregnancies and a high recurrence rate [9].

Early correct diagnosis and timely management of aHUS can significantly improve renal function and overall quality of life [8]. However, the duration, dose, and infusion intervals of Eculizumab therapy remains unclear. Several reports have shown that dose reduction or discontinuation of Eculizumab treatment may lead to TMA relapse, reduce efficacy of treatment, and cause rapid deterioration of vital organs [13]. On the other hand, it has been suggested that Eculizumab can be safely discontinued once clinical remission is achieved and all hematological and renal impairments are normalized, especially in p-aHUS where the trigger is clearly identifiable [7].

After the patient is discharged, it is recommended to monitor the number of reticulocytes in the blood, to perform peripheral blood smears to confirm the absence of schistocytes, to determine the levels of creatinine, lactate dehydrogenase, haptoglobin, as well as complement C3 and C4 as part of regular medical check-ups. The examinations should be performed once a month during the first year after discharge and every 3–6 months afterwards [8].

## Conclusions

p-aHUS is a rare and life-threatening microangiopathic disease which severely impacts maternal and fetal morbidity and mortality. Diagnosis of p-aHUS can be very challenging, as it can mimic the features of various diseases during pregnancy and postpartum. The patient’s outcome can be significantly improved when the condition is discovered and treated immediately. In the last decade, Eculizumab has been the treatment of choice for p-aHUS and has shown promising outcomes for the patients. However, the duration, dose, and infusion intervals of Eculizumab therapy for p-aHUS, as well as the risk assessment of usage in pregnancy remains unclear. More studies are required to determine the optimal regiment to maximize the efficacy of the treatment.
